# AMP-Activated Protein Kinase Suppresses the *In Vitro* and *In Vivo* Proliferation of Hepatocellular Carcinoma

**DOI:** 10.1371/journal.pone.0093256

**Published:** 2014-04-07

**Authors:** Jidong Cheng, Tianliang Huang, Youfeng Li, Yubai Guo, Yuzhang Zhu, Qingjia Wang, Xiaojun Tan, Weisheng Chen, Yongneng Zhang, Weijie Cheng, Tetsuya Yamamoto, Xubin Jing, Jiexiong Huang

**Affiliations:** 1 Department of Internal Medicine, The First Affiliated Hospital of Shantou University Medical College, Shantou, Guangdong, China; 2 Department of Pathology, The First Affiliated Hospital of Shantou University Medical College, Shantou, Guangdong, China; 3 Department of Internal Medicine, Hyogo College of Medicine, Nishinomiya, Hyogo, Japan; Institute of Hepatology - Birkbeck, University of London, United Kingdom

## Abstract

AMP-activated protein kinase (AMPK) is a central metabolic sensor and plays an important role in regulating glucose, lipid and cholesterol metabolism. Therefore, AMPK is a key therapeutic target in diabetes. Recent pilot studies have suggested that diabetes drugs may reduce the risk of cancer by affecting the AMPK pathway. However, the association between AMPK and the proliferation of hepatocellular carcinoma (HCC) is unknown. In this study, we investigated the relationship between AMPK activity and the proliferation of HCC in cell lines, nude mice and human clinic samples. We first investigated the relationship between AMPK activity and cell proliferation in two HCC cell lines, PLC/PRF/5 and HepG2, by two AMPK activators, 5-aminoimidazole-4-carboxamide-1-h-D-ribofuranoside (AICAR) and metformain. AICAR and metformin treatment significantly inhibited the proliferation of HCC cells and induced cell cycle arrest at G1-S checkpoint. We then observed that metformin abrogated the growth of HCC xenografts in nude mice. The clinical pathology of AMPK activity in HCC, including cell proliferation, differential grade, tumor size and microvessel density, was studied by using 30 clinical tissue samples. In HCC tissue samples, phosphorylated AMPK was expressed mainly in cytoplasm. AMPK activity decreased significantly in HCC in comparison with paracancerous liver tissues (P<0.05). AMPK activity was negatively correlated with the level of Ki-67 (a marker of cell proliferation), differential degradation and tumor size (P<0.05), but not with microvessel density, hemorrhage or necrosis in HCC. Our findings suggest that AMPK activity inhibits the proliferation of HCC and AMPK might be an effective target for prevention and treatment of HCC.

## Introduction

AMP-activated protein kinase (AMPK) is highly conserved as a heterotrimer. It contains α1, α2, β1, β2, γ1, γ2 and γ3 subunits with different alternative splicing forms, which results in different combinations of the AMPK complex [1]. AMPK is a sensor of cellular energy status and a regulator of metabolism. It is inactive unless phosphorylated by upstream kinases at a specific threonine residue (Thr-172) within the kinase domain and is sensitive to cellular AMP/ATP ratio, whereby a high AMP or low ATP level activates AMPK [2], [3]. AMPK activation can inhibit anabolic processes such as protein, lipid, or glycogen synthesis by phosphorylating a number of substrates [4]. However, it can also activate catabolic processes such as fatty acid oxidation and glycolysis [5]. Studies have confirmed that AMPK is involved in breast cancer, prostate cancer and lung cancer [6]–[8]. Much of the evidence shows that AMPK might be a therapeutic target for cancer. However, the investigation of the relationship between AMPK and cancer is still in its infancy.

The association between AMPK with several tumor suppressors suggests that therapeutic manipulation of this pathway using established diabetes drugs warrants further investigation in patients with cancer [9]. Liver kinase B1 (LKB1), the upstream activator of AMPK, was previously described as a tumor suppressor gene related to epithelial neoplasia. Loss of function of LKB1 is associated with Peutz-Jeghers syndrome, which is characterized by multiple gastrointestinal polyps and significantly increased lifetime risk of various epithelial cancers, including HCC [10], [11]. LKB1 is both a regulator of gluconeogenesis in hepatocytes and a tumor suppressor gene in epithelial tissues. Recently, LKB1 was found to have a major role in phosphorylating and activating AMPK. In addition, downstream tumor suppressors have been identified [12].

The liver is one of the most important organs associated with digestion, detoxification, production and storage, so the liver has a high metabolic rate, and therefore liver diseases including HCC are associated with metabolic disorders [13]. HCC is one of the leading causes of cancer deaths in the world. However, the exact molecular mechanisms of HCC and effective prevention and treatment are still unclear [14]. To date, the association between AMPK and HCC is unknown. In this study, we investigated the association between AMPK activity and cell proliferation in HCC cell lines and clinical samples of HCC. Our findings suggest that AMPK is involved in cell proliferation in HCC and might be an effective target for prevention and treatment of HCC.

## Materials and Methods

All animal experiments were approved by the Ethics Committee for Animal Experimentation of the Shantou University Medical College. We add written consents from patients or their family members in the last two weeks according to the protocol approved by Shantou University Medical College's human research committee. Because many patients of HCC in this study had passed over, the written consents of these patients were obtained from their family members. Written informed consents was obtained from patients or their family members, and the study protocol conformed to the ethical guidelines of the 1975 Declaration of Helsinki, as reflected in approval by Shantou University Medical College's human research committee.

### Cell lines, cell culture and reagents

The human hepatoma cell lines, PLC/PRF/5 and HepG2, were obtained from the American Type Culture Collection. Cells (2×10^5^ cells) were grown in RPMI1640 (ICN; Biomedicals Inc.) supplemented with 10% fetal calf serum, 100 units/ml penicillin, and 100 units/ml streptomycin (Invitrogen). LKB1 (the upstream activator of AMPK) absent cell line, Hela cells, was obtained from the American Type Culture Collection as a control. 5-aminoimidazole-4-carboxamide-1-h-D-ribofuranoside (AICAR) and 1, 1-dimethylbiguanide hydrochloride (metformin) were purchased from Sigma (St. Louis, MO). Compound C (AMPK Inhibitor) was purchased from Sigma (St. Louis, MO).

### *In vivo* tumor models

BALB/c-nu mice were obtained from SLC (Guangzhou, China). PLC/PRF/5 cells (2×10^6^) were inoculated s.c. in 5-week old male nu/nu mice for 4 points at both flanks (X2). After 1 week, metformin was dissolved in PBS and administered with i.p. injections (30 Ag/g body weight). The control group received vehicle only phosphate buffer saline (PBS). Number and weight of tumors were measured after 7 weeks of treatment. All animal experiments were approved by the Ethics Committee for Animal Experimentation of the Shantou University Medical College.

### Human tissue samples

Tissue samples of cancerous and paracancerous tissues were obtained from 30 patients with HCC undergoing curative hepatectomy (segmental or lobar resection) at the First Hospital Affiliated of Shantou University Medical College between 2005 and 2009. Samples were fixed in 10% neutral formalin and embedded in paraffin for histopathological and immunohistochemistry examination. Fresh samples were stored at −80°C for western blot analysis. Data on alpha fetoprotein (AFP), albumin, globulin, total bilirubin (TBil), alanine amiotransferase (ALT), aspartate aminotransferase (AST), γ-glutamyltranspeptidase (γ-GTP), aspartic acid (ASP), and platelets (PLTs) were obtained from patient hospital records. Tumor size (diameter) was obtained from surgical records. HCC differential grade was classified according to the world health organization classification [15]. Liver fibrosis and hepatitis activity stage were classified according to the New Inuyama Classification [16].

Informed oral consent was obtained from each patient, and the study protocol conformed to the ethical guidelines of the 1975 Declaration of Helsinki, as reflected in approval by our institution's human research committee.

### MTT

Cell proliferation was assessed by the (4, 5-dimethylthiazol-2-yl)-2, diphenyltetrazolium bromide (MTT) method and cell count. Cells were seeded at 4000 cells/well in 96-well plates containing the test compounds for the indicated times and then incubated with 30 µL MTT solution (5 mg/mL in PBS) for 3 h at 37°C. Optical density was determined by an enzyme-linked immunosorbent assay (ELISA) reader.

### Flow cytometry

For cell cycle analysis, cells were treated with or without AICAR or metformin for 1.5 days. Cells (1×10^6^) were trypsinized and fixed in 70% ethanol overnight. Fixed cells were stained with propidium iodide (50 µg/ml) for 30 min at room temperature. Cells were filtered with use of a 5 ml polystyrene round-bottom tube with a cell-strainer cap prior to flow cytometry. Flow cytomentry was performed with a FACSVantage SE cell sorter (Bacton Dickinson). Cell cycle analysis was performed with the ModFit LT software.

### BrdU Incorporation Assay

Cell proliferation was quantified by the measuring the BrdU incorporation during DNA synthesis with BrdU cell proliferation Detection Kit (KeyGen Biotech). The assay was performed according to the manufacturer's manual. In brief, equal number of PLC/PRF5 and HepG2 cells was plated in 24-well plates and serum-starved overnight. Cells were then treated with metformin (10 mM) for 24 minutes, followed by FBS replenishment and BrdU labeling for 1 hours. The BrdU labeling signal was quantified by PE-BrdU antibody and flow cytometry. Each assay was done in triplicate. The experiments were performed at least three times independently.

### RNA Interference

AMPKα1 siRNA was obtained from Santa Cruz Biochnology (sc-270142). A pool of 3 target-specific 19–25 nt siRNAs designed to knock down AMPKα1 gene expression. Cells were inoculated into the 24-mesh board at 37°C, 5% CO2, and saturated humidity conditions up to the convergence rate arriving at 70%–80%. Transfection steps were according to the RNAi manual of Santa Cruz Biotechnology. The proliferation of control, metformin treatment group and siRNA plus metformin treatment group were compared. The proliferation of cancer cell lines was detected by cell count.

### Western blot analysis

Cells and Liver tissues were sonicated in 100 µl RIPA buffer (1×PBS, 1% Nonidet P-40, 0.5% sodium deoxycholate, 0.1% SDS, 100 µg/ml phenyl-methysulforyl fluoride, 45 µg/ml aprotinin, 1 mol sodium orthovanadate), homogenized, and centrifuged (10000 g for 10 min). The supernatant was used for protein determination by use of a BCA Protein Assay Kit (Pierce, IL, United States) and electrophoresis. Samples containing 30 µg proteins were added to SDS-PAGE loading buffer, heated for 3 min at 100°C, loaded onto a gel and then electrophoretically transferred onto polyvinylidene difluoride membrane. The membranes were immunoblotted with anti-phosphor-AMPK antibody (AMPK-p) (Cell signaling Technology, CA, United States), anti-AMPK antibody (Cell signaling Technology, CA, United States), anti-P21^CIP^ antibody (Maixin Bio, Fuzhou, China), anti-p27^KIP^ antibody (Maixin Bio, Fuzhou, China) or anti-cyclin D1 antibody (Maixin Bio, Fuzhou, China), followed by the secondary antibody horseradish peroxidase-conjugated IgG (Vector Laboratories, Burlingame, CA, United States). Signals were developed by chemiluminescence using an enhanced chemiluminescence kit (Amersham, Piscataway, United States). Loading controls of western blot were performed by anti-β-actin antibody.

### Immunohistochemistry

Tissue sections (5-µm thick) were deparafinized with xylene. After dehydration in absolute ethanol 3 times for 3 min each, sections were incubated in 3% (v/v) hydrogen peroxide for 10 min at room temperature, and then washed 3 times for 3 min each in phosphate-buffered saline (PBS; pH 7.4). Tissue sections were microwaved for 20 min in 10 mM citrate buffer (pH 6.0) and washed 3 times in PBS for 5 min each. The paired sections were blocked for 10 min in PBS containing 5% normal serum (1∶50–200 dilution) and then incubated with goat anti-phosphor-AMPK (Cell Signaling Technology), mouse anti-Ki-67 or mouse anti-CD34 (Maixin_bio) at 4°C overnight. Sections were washed twice in PBS and incubated with biotinylated secondary antibody (1∶100) (Vector Laboratories, Burlingame, CA, United States) for 45 min at 37°C. After being washed 3 times in PBS, sections were incubated in streptavidin-peroxidase solution for 30 min at room temperature. The color reaction was performed by 3, 39-diaminobenzidine (DAB) and counterstaining was done by hematoxylin & eosin. The negative control was performed by omitting the primary antibody.

### Semi-quantitative analysis of AMPK activity

The degree of staining was categorized by the extent and intensity of the staining. Three independent observers screened all sections as a semiquantitative evaluation of immunostaining. The immunoreactive score was determined by the sum of the extent and intensity of staining as reported previously [17]. The intensity of staining was scored on a scale of 0 to 3 (0 = negative staining, 1 = weakly positive staining, 2 = moderately positive staining, and 3 = strongly positive staining). The extent of staining (“extent of distribution” of positive cells) was estimated on a scale of 0 to 3 (0 = negative, 1 = positive staining in 1–30% of cells, 2 = positive staining in 30–70%; 3 = positive staining in 70–100%), for a final staining score = (extent score+intensity score)/2.

### Quantization of growth index and microvessel density (MVD)

The percentage of Ki-67-positive cells was calculated as the growth index of HCC. The images of Ki-67 immunostaining by DAB were scanned with use of an Olympus CCD camera and analyzed by Image J (National Institute of Health). The growth index of HCC was calculated as follows: HCC = Ki-67 positive cell number/total cell number in a field. The mean of 5 low-power fields was used.

We assessed MVD as a percentage of the endothelial area as follows: MVD = CD31-positive area/total field area. The images were scanned and analyzed by use of Image J. The mean of 5 low-power fields was used.

### Statistical analysis

Statistical significance was determined by the Kruskal–Wallis test and Kendall rank correlation test (non-parameter statistical methods). The significance level was set at P<0.05.

## Results

**Figure 1:**
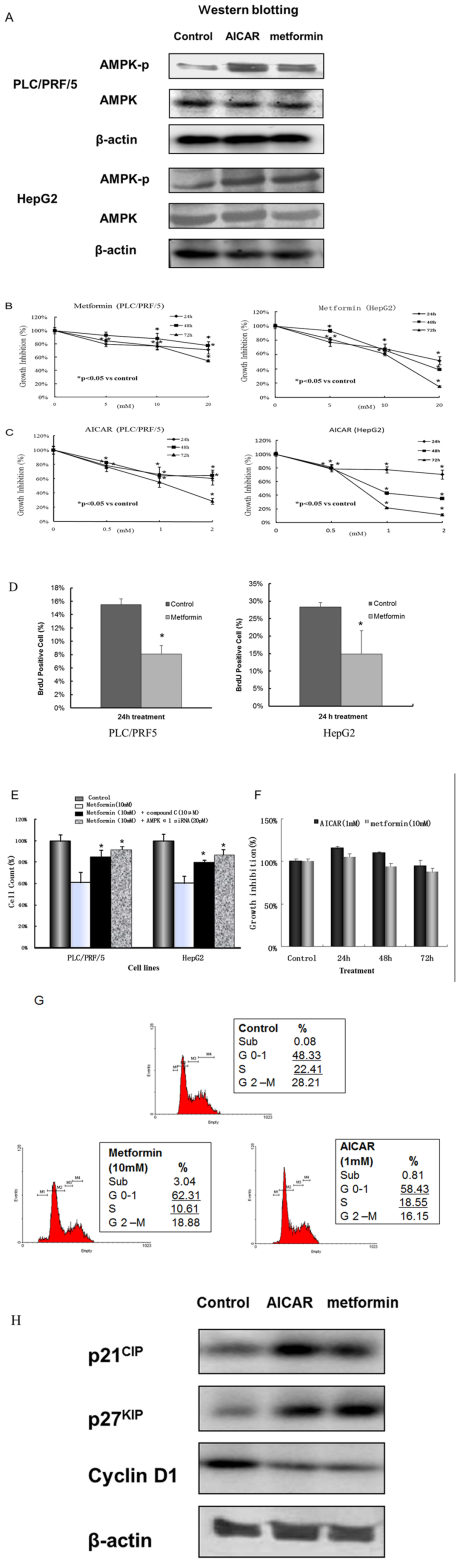
AMPK activity and cell proliferation in HCC cell lines, PLC/PRF/5 and HepG2. (A): Western blot analysis of phosphor-AMPK activation induced by AICAR (1 mM) and metformin (10 mM). Representative bands from three independent experiments. (B, C): Both AICAR and metformin inhibited cell proliferation in a time- and dose-dependent manner (MTT method) in PLC/PRF/5 and HepG2. *p<0.05 (vs. control). (D): BrdU incorporation was significant decreased in both of PLC/PRF/5 and HepG2 cells by metformin treatment for 24 h (E): Metformin inhibited cell proliferation and the growth inhibition was rescued by AMPKα1 siRNA and compound C (an AMPK inhibitor) treatment (cell count method). *p<0.05 (vs. metformin only). (F): As a control, LKB1 (the upstream activator of AMPK) absent cell line, Hela cells did not show growth inhibition with AICAR and metformin treatment. (G): Effect of AMPK activators on cell cycle arrest in HCC cells. Representative DNA histograms for PLC/PRF5 cells are shown. Data from three independent experiments are shown as mean ± SD. (H): Western blot analysis of p21^CIP^, p27^KIP^ and cyclin D1 by AICAR (1 mM) and metformin (10 mM) treatment for 24 hours in PLC/PRF/5 cells.

AMPK activating agents inhibit cell growth and arrest cell cycle in HCC cell lines. To investigate the relationship between AMPK activity and cell proliferation, we used HCC cell lines, PLC/PRF/5 and HepG2, to study the effect of the AMPK-activating agents, AICAR and metformin. Western blot analysis revealed that AMPK could be activated by both AICAR and metformin (Fig. 1A). MTT results indicated that both AICAR and metformin inhibited cell proliferation in a time- and dose-dependent manner (Fig. 1B and C). BrdU incorporation was significant decreased in both of PLC/PRF/5 and HepG2 cells by metformin treatment for 24 hours (Fig. 1D), to imply that DNA synthesis was inhibited. The cell growth inhibition was also further confirmed by cell count method. In addition, the role of AMPK pathway was investigated by silencing AMPK using AMPKα1 siRNA and the pharmacological AMPK inhibitor compound C treatment (Fig. 1E). The growth inhibition of metformin was rescued by AMPKα1 siRNA and compound C treatment. As a control, Hela cells, a LKB1 (the upstream activator of AMPK) absent cell line, did not show cell growth inhibition by AICAR and metformin treatment (Fig. 1F).

We further investigated the mechanism of AICAR and metformin affecting cell proliferation by flow cytometry. Activation of AMPK by AICAR or metformin induced G1 cell cycle arrest in PLC/PRF5 and HepG2 cells. Representative DNA histograms for PLC/PRF5 cells are shown in Figure 1G, which indicated the inhibition of cell proliferation by AMPK. No obvious sub-G0 (apoptosis) peak was observed.

The expressions of cell cycle G1-S checkpoint regulatory proteins, including p21^CIP^, p27^KIP^ and cyclin D1, were further studied by western blot. P21^CIP^ and p27^KIP^ expressions were up-regulated and cyclin D1 expression was inhibited after AICAR and metformin treatment for 24 hours (Fig. 1H). The results suggested that AMPK activity inhibits G1-S checkpoint and induces cell cycle arrest in HCC cells.

### Metformin attenuated the growth of cancer cells in nude mice

**Figure 2:**
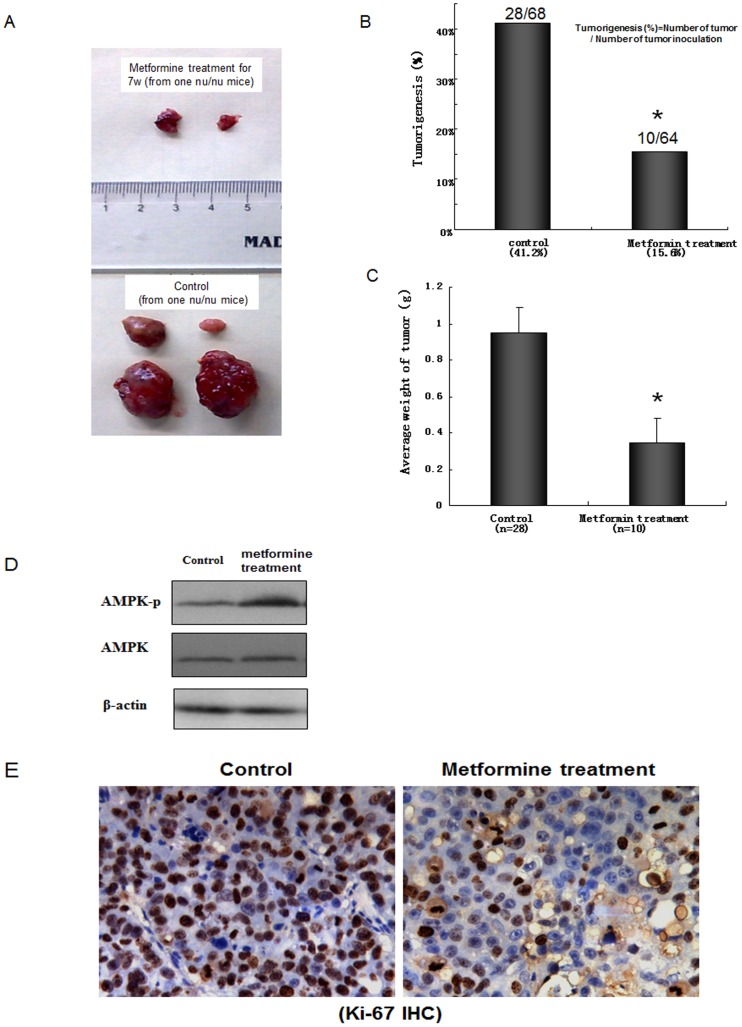
Metformin abrogated the growth of HCC xenografts in nude mice. PLC/PRF/5 cancer cells (2×10^6^) were inoculated s.c. in 5-week old male nu/nu mice at both flanks for 4 points. After 1 week, metformin was dissolved in PBS and given i.p. (30 Ag/g body weight). The control group received PBS only. Control and metformin-treated groups were treated for 7 weeks. (A): Representative picture for tumorigenesis number and size in control and metformin treatment nu/nu mice. (B): The tumorigenesis percentage. (C): Average tumor weight. (D): Western blot analysis of phosphor-AMPK (AMPK-p) and AMPK in control and metformin treated groups. (E): The expression of Ki-67 in control and metformin-treated groups by immunohistochemistry. Data are mean±SD. *p<0.05.

We examined the effects of metformin on the growth of cancer cells *in vivo*. Immunodeficient nude mice were inoculated with PLC/PRF5 cancer cells (s.c.). After the growth of tumors, mice underwent metformin treatment (i.p.). The percentage of tumorigenesis and the average tumor weight of the metformin-treated group were significantly reduced in comparison with that of the vehicle-treated control group (Fig. 2A, 2B, 2C). AMPK activity was up-regulated by metformin treatment (Fig. 2D); whereas, the expression of Ki-67 was decreased by metformin treatment in tumor tissue (Figure 2E). There were no significant differences in body weight and serum glucose level between the control and metformin-treated groups (data not shown).

### AMPK activity in HCC and paracancerous liver tissues

**Figure 3:**
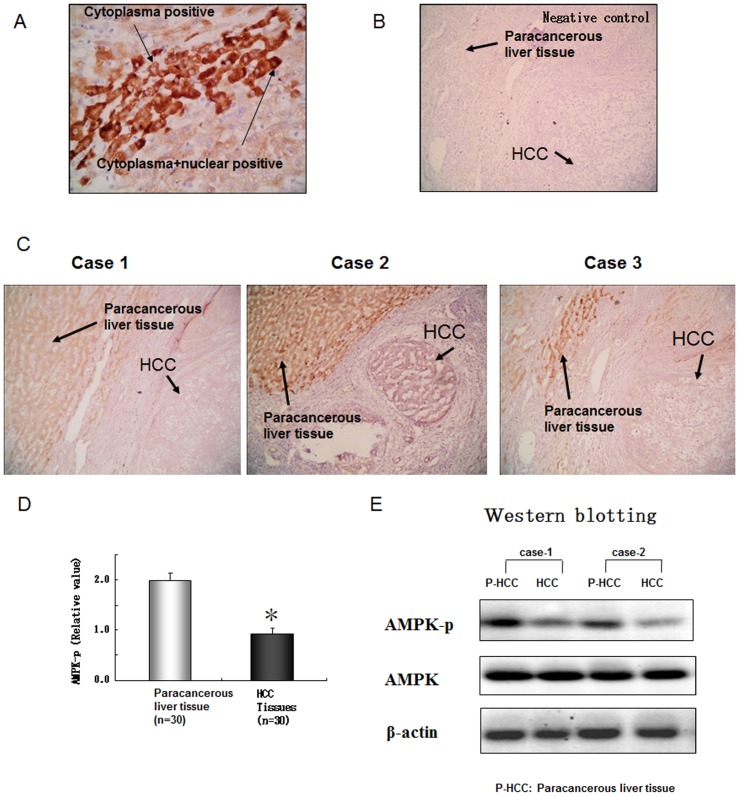
Immunohistochemistry of phosphor-AMPK showed a lower degree of AMPK activity in HCC tissues than in paracancerous liver tissues. (A) Positive signals were detected in cytoplasm and occasionally in nucleus. (B) Negative control (omitting primary antibody). (C)Representative images of three samples of HCC and paracancerous liver tissue showing AMPK activity. (D) Relative AMPK activity in 30 tissue samples. Data are mean±SD. *p<0.05 (E) Western blot analysis of phosphor-AMPK (AMPK-p) and AMPK. Representative bands from five independent experiments.

We investigated AMPK activity in 30 samples of HCC and paracancerous liver tissues by immunohistochemistry and western blot analysis. Immunostaining for AMPK-p was observed in 90% (27/30) of HCC tissues and 93.3% (28/30) of paracancerous liver tissues, mainly in cytoplasm but occasionally in nucleus (Fig. 3A). No positive signal was detected in the negative controls (Fig. 3B). AMPK-p staining was detected in both HCC and paracancerous liver tissues, with weaker staining in HCC tissues (Fig. 3C, 3D). Western blot analysis revealed the level of AMPK activity was significantly lower in HCC than paracancerous tissues (Fig. 3E).

### Correlation of AMPK activity and cell proliferation in HCC

**Figure 4:**
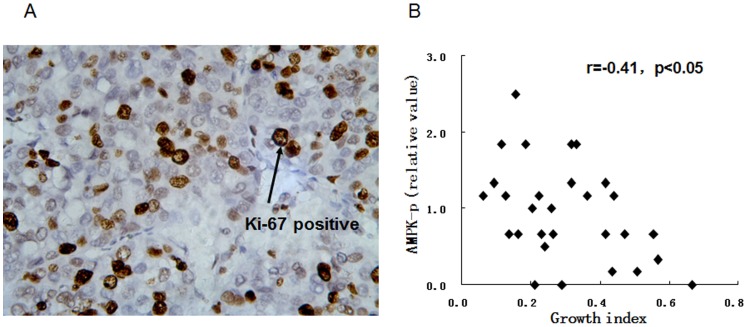
The correlation between AMPK activity and cell proliferation of HCC. (A) The growth index of HCC was represented by the expression of Ki-67. (B) Significant negative correlation between AMPK activity and growth index in HCC (r = −0.41, p<0.05).

The growth index of HCC was determined by the expression of Ki-67. AMPK activity was negatively correlated with the expression of Ki-67 in HCC (r = −0.41, p<0.05) (Fig. 4).

### Correlation of AMPK activity and tumor differential grade in HCC

**Figure 5:**
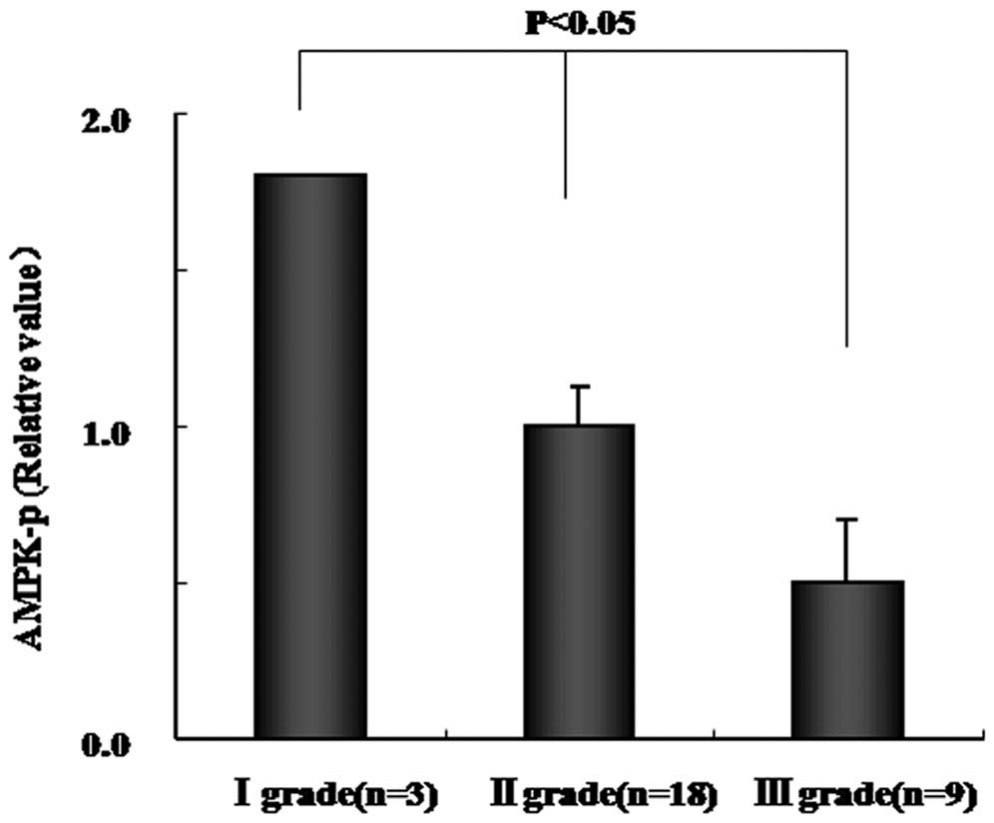
AMPK activity in different degrees of tumor grade in HCC. Data are mean ± SD. P<0.05.

AMPK activity showed a significant down regulation with decreasing differential grade (P<0.05) (Fig. 5).

### Correlation of AMPK activity and tumor size in HCC

**Figure 6:**
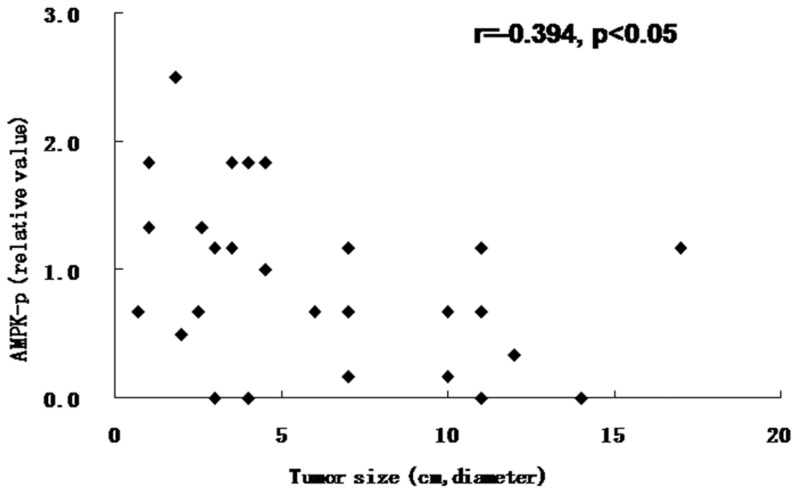
Correlation between AMPK activity and tumor size in HCC (p<0.05).

AMPK activity was negatively correlated with tumor size (diameter) (r = −0.394, p<0.05) (Fig. 6), suggesting that AMPK activity inhibits the proliferation of HCC.

### Correlation between AMPK activity and MVD in HCC

**Figure 7:**
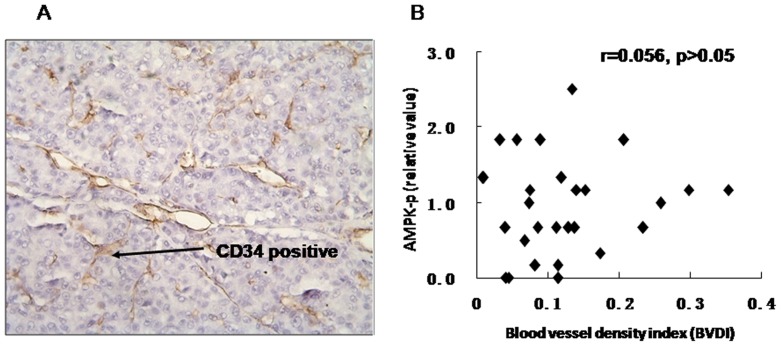
Correlation between AMPK activity and microvessel density (MVD) in HCC. (A) Immunohistochemistry of CD34 level in HCC. (B) Correlation between AMPK activity and MVD (r = 0.056, p>0.05).

HCC is a hypervascular tumor. Angiogenesis is a prerequisite for development and growth in HCC. To study the correlation between angiogenesis and AMPK activity, we analyzed CD34 immunostaining (Fig. 7A) to measure MVD. We found no significant correlation between AMPK activity and MVD (r = 0.056, p>0.05) (Fig. 7B).

### Correlation between AMPK activity and hemorrhage and/or necrosis

As a hypervascular tumor, hemorrhage and necrosis are impartment pathological characters in HCC. However, we found no correlation between AMPK activity and hemorrhage and/or necrosis in HCC tissues (Table 1).

**Table 1:** The correlation between AMPK-p and hemorrhage and/or necrosis in HCC.

Groups	(+/−)	N	AMPK-p relative value	p value
Hemorrhage	(+)	6	0.9±0.4	0.860
	(−)	24	0.9±0.7	
Necrosis	(+)	12	1.2±0.7	0.151
	(−)	18	0.8±0.5	
Hemorrhage or Necrosis	(+)	15	1.1±0.7	0.134
	(−)	15	0.7±0.6	

AMPK: AMP-activated protein kinase. HCC: hepatocellular carcinoma.

### Correlation between clinical features and activation of AMPK

**Figure 8:**
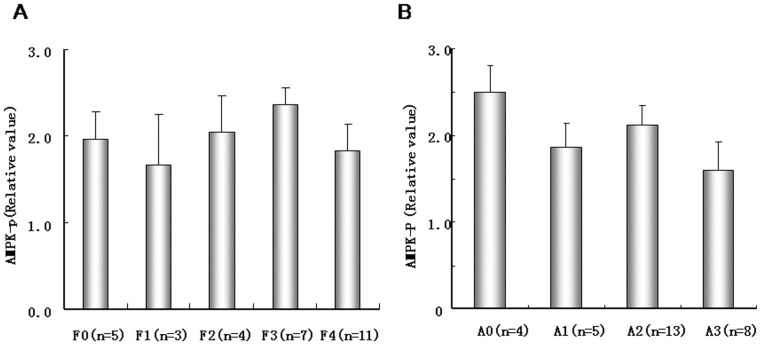
Degree of liver fibrosis (A) and hepatitis activity (B) in paracancerous liver tissues. Degree of liver fibrosis and hepatitis activity degree was classified according to the New Inuyama Classification. Data are mean±SD.

The correlation between AMPK activity and clinical data, including the levels of AFP, albumin, globulin, TBil, ALT, AST, r-GTP, ASP,and PLTs, are shown in Table 2. A significant correlation was only observed between AMPK activity and globulin level (p<0.05). To investigate whether liver fibrosis and hepatitis activity affect AMPK activity in paracancerous tissues, we compared AMPK activity in different liver fibrosis and hepatitis activity grades in paracancerous tissues. We found no correlation between the degree of liver fibrosis and hepatitis activity with AMPK (Fig. 8A, 8B).

**Table 2:** The correlation between AMPK-p and the clinical features of HCC patients.

Clinical Feature	n	rs value	P value
Age	30	0.32	0.088
AFP	23	−0.36	0.093
Albumin	28	−0.24	0.220
Globulin	28	−0.4	0.030*
TBiL	28	0.21	0.286
ALT	28	0.01	0.972
AST	28	−0.21	0.293
γ-GTP	28	−0.24	0.226
ALP	28	0.14	0.489
PLT	28	−0.10	0.598

*P<0.05.

AMPK: AMP-activated protein kinase. HCC: hepatocellular carcinoma. AFP: lpha fetoprotein. TBil: total bilirubin. ALT: alanine amiotransferase. AST: aspartate aminotransferase. γ-GTP: γ-glutamyltranspeptidase. ALP: alkaline phosphatase. PLT: platelet.

## Discussion

In this study, we investigated the relationship between AMPK activity and HCC proliferation in cell lines, animal model and clinical samples. In *in vitro* study two AMPK activators, AICAR and metformin, inhibited the proliferation and induced cell cycle arrest in the HCC cell lines, PLC/PRF/5 and HepG2. In *in vivo* study metformin attenuated the growth of cancer cells in nude mice. Furthermore, in clinical HCC tissue samples, we found lower AMPK activity in HCC than in paracancerous liver tissues. AMPK activity was negatively correlated with cell growth, tumor differential grade and tumor size in HCC, indicating that AMPK activity might have a suppressive effect on HCC. Our findings suggest that AMPK activity is negatively correlated with cell proliferation and induces growth inhibition in HCC. AMPK could be a therapeutic target for HCC.

AMPK is a heterotrimeric complex composed of a catalytic α subunit and regulatory β and γ subunits, each of which is encoded by 2 or 3 distinct genes [18]. AMPK is activated by increases in AMP/ATP ratio caused by cellular and environmental stress, such as hypoxia, ischemia and heat shock [19]. Studies have shown that AMPK regulates the metabolism of fatty acids and glycogen, protein synthesis and cell proliferation. AMPK regulates multiple metabolic pathways through direct phosphorylation of substrates [20]. The proper regulation of AMPK is highly relevant to metabolic disorders such as obesity and diabetes, in which AMPK functions at multiple steps. As a sensor of cellular energy status and a regulator of metabolism, AMPK regulates multiple metabolic processes inside the cell and can be considered as a potential candidate for the metabolic switch from normal to malignant growth [21], [22]. Malignant tumors, including HCC, represent a fundamental metabolic difference between cancer and normal cells. Cancer cells use glucose at a higher rate than do normal cells and metabolize glucose mainly to lactate rather than through mitochondrial oxidative phosphorylation to produce ATP under normal oxygen levels, even though the ATP productivity rate is much lower [23]. Elevated cellular AMP level activates AMPK to inhibit ATP-consuming anabolic pathways and activate ATP-generating catabolic pathways to maintain cellular energy homeostasis [24]. Recent studies show that metformin, an AMPK activator, inhibits HCC cell growth in vitro by inducing cell cycle arrest and decreases the risk of HCC in the general population [25], [26]. In this study, we found a lower AMPK activity in HCC than in paracancerous liver tissues, suggesting that the loss of AMPK activity is an important event in the development of HCC. We found that AMPK activity was related to cell growth and differentiation in HCC cell lines and clinical samples, suggesting that AMPK is an important regulator of HCC proliferation and differentiation, through its mechanism in cellular energy homeostasis in HCC needs further study.

Having determined that tumor progression is inversely related to AMPK activity, we tested whether pharmacological activators of AMPK could suppress HCC. MTT, cell count and flow cytometry indicated that AICAR and metformin inhibited cell growth, which suggests that AMPK plays a significant role in the regulation of cell proliferation in HCC cells. In HCC cells,AICAR and metformin treatment might activate AMPK via an LKB1–AMPK pathway, thus resulting in activation of some or even all of the downstream pathways for proliferation-inhibitory and anti-tumor effects [27]. These results provide solid evidence that AMPK activators can delay the growth of HCC. Metformin has been in clinical treatment for type 2 diabetes for many years and is well tolerated, so it could be tested immediately in cancer patients.

Of note, LKB1 is a well-recognized tumor suppressor, and mutations in the gene encoding LKB1 cause the rare inherited Peutz-Jeghers syndrome. The LKB1–AMPK pathway may function as a cellular energy-sensing checkpoint that controls cell growth and proliferation according to the availability of fuel supplies [28]. The tumor-suppressor LKB1 is upstream of AMPK and is a serine/threonine protein kinase. LKB1 can suppress cell growth and induce a G1 cell cycle arrest, indicating its proliferation-inhibitory and anti-cancer effects [29]. Our results suggest that the LKB1-AMPK pathway can effect the proliferation of HCC.

AMPK activation by AICAR and metformin suppressed cell growth by inducing a G0-G1 cell cycle arrest via regulating p21^CIP^, p27^KIP^ and cyclin D1 in HCC in this study. It has been shown that AMPK inhibits cell-cycle progression by controlling the phosphorylation and stability of p27kip, a cell-cycle inhibitor. The stabilization of p27^KIP^ has been postulated to enable cancer cells survive better under conditions of nutrient and energy stress [30]. Other potential AMPK anti-cancer targets include p53, which supposedly, following phosphorylation by AMPK, induces apoptotic cell death rather than enhancing survival [31]. AMPK activation by AICAR has also been recently reported to inhibit proliferation of various cancer cell lines *in vitro* and *in vivo* by increasing p21^CIP^, p27^KIP^ and p53 [32]–[34]. The S phase cell reduction may be an important event in cell cycle arrest induced in HCC cell lines by AMPK activators. Future studies need to address how AMPK activator inhibits HCC cells in crossing the G1/S boundary.

Angiogenesis is a prerequisite for development and growth of different human tumors. For HCC, only a few studies have described the mechanisms of microvessel formation [35]. No report has described the relationship between angiogenesis and AMPK activity in HCC. In both experimental and clinical studies, AMPK induces angiogenesis. In this study, we did not find AMPK activity correlated with MVD in HCC tissues. However, our results are limited in showing the correlation of AMPK activity in HCC cells and angiogenesis, and the relationship between AMPK activity in microvessels of HCC and angiogenesis needs further study.

We found no significant correlation between AMPK activity and hemorrhage and/or necrosis, or between AMPK activity and clinical features, including levels of AFP, albumin, TBil, ALT, AST, r-GTP, ASP, or PLT, except for globulin. In addition, we found no effect of AMPK activity on degrees of liver fibrosis and hepatitis activity in paracancerous liver tissues. Therefore, AMPK activity could be independent of other clinical pathological features during the development of HCC.

In this study, we demonstrate for the first time in cell lines and HCC tissue samples that AMPK and AMPK-activating drugs traditionally used to counter the metabolic changes observed in diabetes could be effective in restraining HCC cell proliferation. AMPK might inhibit HCC by regulating metabolism and the cell cycle, so the AMPK system might be a great therapeutic option for HCC.
